# Understanding implicit and explicit sensorimotor learning through neural dynamics

**DOI:** 10.3389/fncom.2022.960569

**Published:** 2022-08-03

**Authors:** Xueqian Deng, Mengzhan Liufu, Jingyue Xu, Chen Yang, Zina Li, Juan Chen

**Affiliations:** ^1^Center for Studies of Psychological Application, Guangdong Key Laboratory of Mental Health and Cognitive Science, School of Psychology, South China Normal University, Guangzhou, China; ^2^Key Laboratory of Brain, Cognition and Education Sciences (South China Normal University), Ministry of Education, Guangzhou, China; ^3^Department of Neurology, Johns Hopkins University, Baltimore, MD, United States; ^4^Institute for Mind and Biology, The University of Chicago, Chicago, IL, United States; ^5^Department of Cognitive Science, University of California San Diego, La Jolla, CA, United States

**Keywords:** sensorimotor learning, explicit, implicit, neural dynamics, motor learning

## Introduction

The field of sensorimotor learning investigated people's ability to adjust its movements in face of sensory perturbations (Cunningham, 1989; Hwang et al., 2006; Telgen et al., 2014; Kim et al., 2021). A bulk of behavioral paradigms strongly support that sensorimotor learning has two distinct components: an explicit component in which participants are aware of the perturbations and would employ strategies, and an implicit component where the motor controller implicitly adapts itself (Benson et al., 2011; Huberdeau et al., 2015). One canonical phenomenon of sensorimotor learning is that humans are explicitly aware of the sensory perturbations but do not seem to have voluntary control over their behavioral learning; in some cases, implicit adaptation would even act against the participants' explicit strategies (Mazzoni and Krakauer, 2006; Taylor and Ivry, 2012; Hadjiosif et al., 2021; Yang et al., 2021).

One possible way to interpret sensorimotor learning and explain its puzzling phenomena is through a neural dynamics framework (Shenoy et al., 2013; Kaufman et al., 2014; Vyas et al., 2020; Barack and Krakauer, 2021; Sohn et al., 2021). We will navigate through the concepts of sensorimotor learning and neural dynamics, and propose a neural dynamics framework for the explanandum.

## Explicit and implicit systems of sensorimotor learning

In a canonical experiment of visuomotor rotation, participants were instructed to counter the imposed perturbation using an explicit strategy in a pointing task (Mazzoni and Krakauer, 2006). They were told exactly of the nature of perturbation: a 45° counterclockwise rotation from the desired target. Soon after the onset of perturbation, the participants realized it and employed the following explicit strategy: aim for the direction 45° clockwise to the presented target. The strategy initially works perfectly and reduces performance error to nearly zero, but surprisingly performance keeps deteriorating as the experiment continues. Moreover, the amount of learning, quantified as the directional difference between the reach directions for the same target at the start and the end of learning, was the same for the experiment group and the control group (the experiment group uses explicit strategy, and the control group is not allowed to do so). While sensorimotor learning improved task performance for the control group, it deteriorated performance for the experiment group. The results strongly suggest the existence of an implicit learning system that is distinct from the explicit system we are commonly aware of during sensorimotor perturbation.

By incorporating catch trials, Benson et al. showed that the employment of explicit strategy decreased the degree of implicit learning throughout trials and led to a decreased aftereffect at the end (Benson et al., 2011). In a recent study of manual tracking tasks, Yang et al. show that humans were able to rapidly build a new controller for movement *de novo* when facing a completely different operational environment. The results also suggest that participants do not rely on any explicit processes to establish the new controller (Yang et al., 2021).

To better characterize the two components, Taylor et al. quantified the exact contributions of explicit and implicit learning in a visuomotor rotation experiment (Taylor et al., 2014). They employed a novel task design that allows them to probe the explicit aiming directions of the participants. In each trial, participants first indicated the aiming direction they planned before the actual reaching. As such, the experimenters were able to assess the explicit aiming directions and actual reaching directions, which corresponded to explicit learning and implicit learning, respectively. The results show remarkable time course differences between implicit and explicit learning: while explicit learning was rapid and large at the beginning, implicit learning was slow and monotonic. Continuous error feedback increased the contribution of implicit learning to overall learning over time. McDougle and Taylor later show that the explicit component of sensorimotor learning involves not a single but many different cognitive strategies throughout the course of sensorimotor learning (McDougle and Taylor, 2019).

A recent study by Miyamoto et al. provides interesting insight into why humans perhaps need two systems for sensorimotor learning. The task design allowed experimenters to probe into the interactions between implicit and explicit learning in both perturbation-driven and perturbation-free conditions. Before each reaching, the participants were asked to indicate their aiming strategy by positioning a marker. The explicit learning component is operationally defined as the difference between the marker and the cursor target, while the implicit learning component was defined as what's leftover. The trial sequence was composed of sine-shaped components at different frequencies. The results showed that implicit and explicit learning synergize in drive dimensions and cancel each other in undriven dimensions, and the cancellation happens since implicit learning is compensating the noises of low-fidelity explicit learning (Miyamoto et al., 2020).

In short, numerous studies have shown that there are two distinct systems under sensorimotor learning: an explicit strategy learning system and an implicit adaptation learning system (Funahashi et al., 1997; Jueptner et al., 1997; Hoshi et al., 2000; Tanji and Hoshi, 2008; Lee et al., 2020). The intriguing question followed would be what the neural underpinnings of such characteristics of human sensorimotor learning could be.

## Understanding sensorimotor learning through neural dynamics

There is a growing trend in current neuroscience to use methods of dynamics and manifolds to understand possible mechanisms of neural representation and computation, and motor neuroscience particularly is a field where neural dynamics has offered valuable insights (Shenoy et al., 2013; Kaufman et al., 2014; Abbott et al., 2016; Vyas et al., 2020).

The brain cortex is a system of interconnected neurons, and its activity can be described with two classes of features: the firing of individual neurons, and the biochemical structure defined by cell membrane properties, intracellular fluid, synaptic strength, and so forth. While neuronal firing takes place and changes over a short time scale, the latter generally changes much less rapidly. Alternative to the connectionist view of neuron groups as circuits, the perspective of the neural dynamics sees the spiking activity (probability) of each neuron at a particular moment as one dimension of a vector. Then, the neural ensemble as a whole has a position in high dimensional state space at any moment, and across time the shifting positions form a continuous trajectory of state evolution with time as a latent variable.

The non-spiking features of a neuron group entail that these trajectories cannot possibly reach the whole state space, because the synaptic weights between neurons make certain spiking profiles physiologically impossible. Therefore, in the steady stage, a neuron ensemble only lives in a subspace or manifold in the whole space (Barack and Krakauer, 2021). It has been shown with various cognitive and motor processes that neural trajectories follow stereotyped patterns or shapes embedded in the manifolds (Kaufman et al., 2014; Sohn et al., 2019; Vyas et al., 2020). What's also observed is that trajectories are clustered into distinct groups, either significantly separated from each other or even progressing toward opposite directions in the state space. In different experiment tasks, such clustering stably encodes different task variables including motion strength, movement direction, and motor memories, etc. (Kaufman et al., 2014; Bachschmid-Romano et al., 2022; Sun et al., 2022). The clusters of trajectories also have distinct clusters of initial points, so for a particular trial its trajectory can be reliably predicted by the initial state of the neural ensemble (Kaufman et al., 2014; Sohn et al., 2019).

We hypothesize that the explicit component of sensorimotor learning toggles the initial point of neural trajectory and tries different existing movements without reshaping the manifold. The implicit component, on the other hand, changes the synaptic weights among neurons and reshapes the manifold. Implicit learning is a slow and gradual process characterized by persistent aftereffects (Mazzoni and Krakauer, 2006; Hadjiosif et al., 2021)). These characteristics well-correspond to the change of synaptic weights and manifold shape: biochemical processes are relatively slow, and their effects cannot be quickly undone. In the implicit learning paradigms, the aftereffects themselves suggest that implicit learning creates a novel mapping between sensory goals and motor commands, and in the perspective of the neural dynamics, that would require the manifold itself to be adapted. Though few studies had directly addressed implicit adaptation with neural dynamics, the reassociation between behavior and neural activity and the reorganization between preparation-related activity and movement-related activity have been well-characterized by state-space models (Rokni et al., 2007; Elsayed et al., 2016; Golub et al., 2018; Sauerbrei et al., 2020). The high similarity between such reassociation and the observations of implicit adaptation may imply a similar or even common neural basis.

The absence of lasting aftereffects for explicit learning and its relatively rapid time scale imply that it doesn't involve a permanent change of manifold shape and patterns of neural trajectories. In our framework, we propose that explicit learning sets the initial point of neural trajectory for movement, and by setting different initial points in the state space, one is able to explicitly learn the sensorimotor task in a trial-and-error manner. Motor preparation studies support that cortical regions upstream to M1 send direction-specific inputs to the M1 manifold to generate a movement in that particular direction by setting the initial conditions for the dynamics of the network (Shenoy et al., 2013; Kao et al., 2021; Bachschmid-Romano et al., 2022). Explicit sensorimotor learning boils down to re-aiming strategies that explicitly select existing motor plans, and it is likely that motor preparation and explicit learning share a similar or the same neural basis. Therefore, the way explicit learning interacts with the M1 manifold can well be the selection of initial points. The prefrontal cortex is likely the neural correlate of explicit learning (Hoshi et al., 2000; Tanji and Hoshi, 2008; Goto et al., 2011; Ono et al., 2015). During the course of a visuomotor rotation task, as we shall see that, at the very beginning, the neuronal activity in the prefrontal cortex tries out different initial points on the manifold to try to solve the task, which is known as the explicit system of sensorimotor learning; then, as the learning continues, the synaptic weights and thus the manifold are modified by the implicit system in the sensorimotor learning (Matsumoto et al., 2003; Graydon et al., 2005; Narayanan and Laubach, 2006; Levy and Wagner, 2011).

Here we give an exemplary prediction in neural dynamics framework in the Mazzoni et al. experiment. It's a very speculative one for the purpose of showing how concrete predictions could be raised under the language of neural dynamics framework. Mazzoni et al. observed that in the early stage of learning the visuomotor rotation task, participants were able to solve the task perfectly with explicit re-aiming, but as learning progressed their explicit strategy was overridden by its implicit counterpart (Mazzoni and Krakauer, 2006). Previous studies showed that a highly possible neural correlate of explicit motor learning is the prefrontal cortex (Pascual-Leone et al., 1996; Matsumoto et al., 2003; Anguera et al., 2010; Liew et al., 2018; Lee et al., 2020). Thus, a best first-hand guess is to place the electrode recording arrays in prefrontal cortex to investigate explicit learning. For implicit learning, cerebellum has been long suggested as the neural correlate (Galea et al., 2010; Liew et al., 2018). Recently, neural dynamics analysis has been performed in other motor tasks for Purkinje cells in cerebellum (Zobeiri and Cullen, 2022). Thus, it's reasonable to investigate our cerebellum cells with neural dynamics first. Neural recording shall be taken throughout the course of reproduction of Mazzoni et al. experiment. Then there could be constructed a highly dimensional neural dynamics space by taking every neuron activity as a single dimension of the space. Then, for each trial of visuomotor adaption, a neural trajectory in this highly dimensional space could be constructed taking all individual neural activities as dimensions. At the early phase, we hypothesize: when it's mostly explicit learning at work, we shall see the neural trajectory is taking different initial points but very similar trajectories, as we explained in the previous paragraph, explicit learning is easier and more variable and in manifolds it's easier to take different initial points rather than altering the whole trajectories. However, as in the late phase where implicit learning is taking over explicit learning, we shall see neural trajectories taking very close initial points but every different trajectories in the highly dimensional space. This example is a speculative but specific one of how a hypothesis could be risen under the framework of neural dynamics. Fruitful progress could be yielded utilizing this new framework re-investigating behavioral experiments of implicit-explicit motor learning.

While there is a general consensus of the loci of explicit learning, the loci of implicit learning are still under debates until today. Cerebellum has been thought to be the most potential candidate (Galea et al., 2010; Butcher et al., 2017). However, more recent tDCS study shows no effects of stimulating cerebellum on motor learning in visuomotor rotation tasks (Liew et al., 2018). It's possible that the rich neural dynamical processes in cerebellum might not survive through global stimulation like tDCS (Zobeiri and Cullen, 2022). Thus, it's important if cerebellum studies of motor learning could incorporate framework of neural dynamics. Also, there is also a rich repertoire of implicit processes in the cortices for sensorimotor control, which potentially underlies the implicit component of sensorimotor learning. A fronto-basal-ganglia circuit is an underlying neural correlate for “dorsal pathway” natural grasping motor control (Milner and Goodale, 2006; Prabhu et al., 2007). Another fronto-parietal circuit is the underlying mechanism for motor stop-signal control (Verbruggen and Logan, 2008). While more research are needed to better understand the relation between sensorimotor learning and sensorimotor control, the neural dynamics framework does not limit from the problem of limit understanding; instead, it's here to yield more understanding. Neural dynamics analysis potentially could place sensorimotor control and sensorimotor learning into the same analysis space, since, at the end of the day, there are the same group of neurons firing just in different patterns. Further analysis could be done on comparing the neural dynamics under sensorimotor control tasks and neural dynamics under sensorimotor learning tasks. Therefore, neural dynamics framework could be also very useful to learn about the underlying neural correlate of implicit learning, and relation between sensorimotor learning and sensorimotor control.

In short, we review literature from behavioral studies of implicit-explicit motor learning and electrophysiology studies of neural dynamics. We provide examples of how future research could combine the two lines of inquiries, and suggest how potential fruitful results could be yielded.

## Author contributions

XD and ML drafted the manuscript. CY, JX, and ZL provided feedback on the manuscript. JC edited the final manuscript. All authors approved for the final version of the manuscript.

## Funding

This research was supported by the National Natural Science Foundation of China (No. 31970981) and the National Science and Technology Innovation 2030 Major Program (No. 2022ZD0204802).

## Conflict of interest

The authors declare that the research was conducted in the absence of any commercial or financial relationships that could be construed as a potential conflict of interest.

## Publisher's note

All claims expressed in this article are solely those of the authors and do not necessarily represent those of their affiliated organizations, or those of the publisher, the editors and the reviewers. Any product that may be evaluated in this article, or claim that may be made by its manufacturer, is not guaranteed or endorsed by the publisher.
